# Paving the Way for Electronic Patient-Centered Measurement in Team-Based Primary Care: Integrated Knowledge Translation Approach

**DOI:** 10.2196/33584

**Published:** 2022-03-18

**Authors:** Selena Davis, Marcy Antonio, Mindy Smith, Paul Burgener, Danielle C Lavallee, Morgan Price, Sarah C Fletcher, Francis Lau

**Affiliations:** 1 School of Health Information Science University of Victoria Victoria, BC Canada; 2 School of Information University of Michigan Ann Arbor, MI United States; 3 College of Human Medicine Michigan State University Grand Rapids, MI United States; 4 Patient Advisory Committee Kootenay Boundary Collaborative Services Committee Castlegar, BC Canada; 5 Patient Voices Network BC Patient Safety & Quality Council Vancouver, BC Canada; 6 Michael Smith Health Research BC Vancouver, BC Canada; 7 Innovation and Support Unit Department of Family Practice, Faculty of Medicine University of British Columbia Vancouver, BC Canada

**Keywords:** patient-centered measurement, patient-centered care, primary health care, team-based care, knowledge translation, patient-oriented research, patient portals, patient-generated health data, patient-reported experience measures, patient-reported outcome measures, rural health services, patient data, digital health, electronic data, patient portal, mental health

## Abstract

**Background:**

Patient-centered measurement (PCM) aims to improve the overall quality of care through the collection and sharing of patient values, outcomes, and perspectives. However, the use of PCM in care team decisions remains limited. Integrated knowledge translation (IKT) offers a collaborative, adaptive approach to explore best practices for incorporating PCM into primary care practices by involving knowledge users, including patients and providers, in the exploratory process.

**Objective:**

This study aims to test the feasibility of using patient-generated data in team-based care; describe the use of these data for team-based mental health care; and summarize patient and provider care experiences with PCM.

**Methods:**

We conducted a multi-method exploratory study in a rural team-based primary care clinic using IKT to co-design, implement, and evaluate the use of PCM in team-based mental health care. Care pathways, workflows, and quality improvement activities were adjusted iteratively to improve integration efforts. Patient and provider experiences were evaluated using individual interviews relating to the use of PCM and patient portals in practice. All meeting notes, interview summaries, and emails were analyzed to create a narrative evaluation.

**Results:**

During co-design, a care workflow was developed to incorporate electronically collected patient-generated data from the patient portal into the electronic medical record, and customized educational tools and resources were added. During implementation, care pathways and patient workflows for PCM were developed. Patients found portal use easy, educational, and validating, but data entries were not used during care visits. Providers saw the portal as extra work, and the lack of portal and electronic medical record integration was a major barrier. The IKT approach was invaluable for addressing workflow changes and understanding the ongoing barriers to PCM use and quality improvement.

**Conclusions:**

Although the culture toward using PCM is changing, the use of PCM during care has not been successful. Patients felt validated and supported through portal use and could be empowered to bring these data to their visits. Training, modeling, and adaptable PCM methods are required before PCM can be integrated into routine care.

## Introduction

### Background

Seminal work in the 1960s and 1970s, supporting the combination of the concepts of a medical home with attributes of patient-centered care [1], provided the foundation for research that demonstrated the benefits of this care model (eg, comprehensive, coordinated care within a primary care team). Benefits linked to patient-centered medical home models include improved health-related quality of life, self-management, and depression; reduced hospital admissions; and improved clinical measures such as blood pressure and glycated hemoglobin [2]. However, the concept of patient-centeredness has been poorly theorized and operationalized, although several papers have identified key attributes such as patient and family being respected; given complete health information; being involved in decision-making; and supported in their physical, psychological, and social needs [3,4]. Newly realized in British Columbia (BC), the patient’s medical home is a community practice that operates at an ideal level to provide longitudinal patient-centered, team-based primary care [5].

To assess these attributes, measures are needed to capture patient-centered care in a form that can be used to rate care quality and quality improvement (QI). Patient-centered measurement (PCM) has the potential to capture data to improve patient care experiences, care quality, communication, and trust. To this end, the BC Ministry of Health and the 7 health authorities established the BC PCM Steering Committee [6] in 2021 to implement scientifically rigorous approaches to collect and report patient-generated data (PGD). Examples of sources of these data include patient-reported experience measures (PREMs) and patient-reported outcome measures (PROMs).

However, the integration of PCM into regular care visits and decisions presents major conceptual, methodological, and logistical challenges in translating this body of knowledge into routine clinical practice [7-9]. A systematic review found that PGD improved patient health awareness and communication with providers but that difficulties arose when patients wanted greater provider involvement with their data during clinic visits [10]. Lordon et al [10] found that providers had difficulty accommodating patient requests for engagement with PGD because of the perceived lack of value, time constraints, and lack of workflow integration. Even with access to patient portals for data entry, the ability to incorporate and track these data varies across systems because of organizational, practice, workflow, resource, and technological challenges [11-13].

There is an unprecedented opportunity to develop and test best practices for incorporating PCM into clinical care because of the rapid uptake of virtual care and enhanced digital literacy for both patients and providers during the COVID-19 pandemic. This requires planning to determine which strategies to use, selection considerations about which patient populations to target for PREMs and PROMs, choice of specific measures, and engagement considerations, including how clinical care teams will incorporate and act on the data [14]. Given the complexity of implementing PCM in clinical practice, multilevel implementation science frameworks are effective, with the choice of framework or theory based on *fit for purpose* [15,16]. Iterative knowledge sharing, or integrated knowledge translation (IKT), was identified for our research, whereby planning, implementing, and evaluating team-based care performance could be optimally developed through the lens of providers, patients, and the research team [17]. Synergies derived from this IKT approach enhance the understanding of patients’ and providers’ context and needs, thereby enhancing the relevance of the generated research and increasing user knowledge and understanding of the research process, awareness of the research, and appreciation for how and when it can be applied [18].

For this research, we partnered with the regional district of Kootenay Boundary (KB) Division of Family Practice [19], whose mission is to help rural practitioners meet patient and practice needs and lead change as part of a province-wide initiative to strengthen health care in BC. The focus on people living in rural and remote communities is critical to address limited access to providers and services, and the resulting health disparities of higher chronic disease multimorbidity and all-cause mortality [20-22]. In this context, digital health solutions have great potential to address these health disparities [23]. The study team assessed priority areas for rural clinical care and identified mental health care as a high-needs area based on BC Community Health Data [24]. Mental illness is a common and disabling health problem in Canada, affecting 1 in 5 Canadians, and has become of even greater importance during the COVID-19 pandemic [25]. Care team–based patient-centered planning strategies have important potential in the treatment of mental illnesses, such as anxiety and depression [26]. Mental health concerns have worsened during the pandemic; based on the 2019 Community Health Survey, almost 5 million Canadians aged ≥12 years (16%) had seen or spoken to a health care professional in the previous year about their mental health, an increase of 2% since 2015 [27].

### Objectives

The overall research aim was to develop new methods to incorporate patient-generated mental health and experience data for team-based in-clinic and virtual care. The new methods for PCM that emerged from this study are reported in another publication (M Antonio et al, unpublished data, December 2021). This paper reports on the IKT approach that was used in the multi-method study with the following objectives: (1) to explore the feasibility of integrating PGD using a patient portal in team-based care; (2) to describe the use of these data for team-based mental health care; and (3) to summarize patient and provider care experiences with PCM.

## Methods

### Setting

The study took place in a rural team-based care practice in the southern interior of BC, Canada, between February 2020 and April 2021. The private, multi-provider primary care clinic served as the patient’s medical home [5]. The study received institutional ethical approval (protocol number: BC H19-03855). Notably, the first stage began at the onset of the COVID-19 pandemic, and the study was completed during the pandemic. As such, the research was conducted virtually, including regular interactions with providers, patients, and the research team.

### Study Design

**Figure 1:**
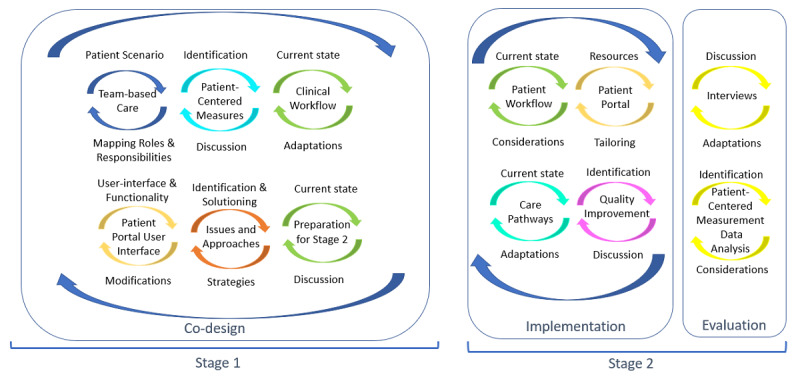
Multi-method, integrated knowledge translation approach for the integration of patient-generated data.

The research used a multi-method, IKT approach with interconnected stages of study (Figure 1). In line with the directive in a scoping review on IKT in health care [28], we created a protocol, timeline, and IKT plan to guide our efforts, and the analysis of continuous and documented feedback offered the research team the ability to report findings with sufficient detail to reveal how IKT was associated with outcomes. Stage 1 of the study involved co-design during which the research team worked with the care team to identify relevant PCMs and optimize the use of the patient portal in the context of the team’s clinical needs, roles and responsibilities, and workflow. Stage 2 included the implementation of the portal and care workflows with adaptation based on feedback and evaluation of its use and impact on clinical care. Implementation and evaluation occurred concurrently so that feedback could be incorporated and further evaluated.

The research team included patient partners, researchers, subject-matter expert scholars, and our industry partner. The patient partners (PB and MS) and two researchers (MA and SD) met regularly with clinic staff and care providers recruited to the study and with other knowledge users at various points within the study, including local professionals from our study collaborators, KB Division of Family Practice and General Practice Service Committee Practice Support Program [29]. Monthly research meetings were held with the entire research team to discuss and apply what we had learned to date.

### Recruitment and Composition of the Provider Practice

A pragmatic approach and convenience sampling were used to recruit a community team-based clinic. The KB Division of Family Practice invited one of the early adopter clinics of a patient medical home care model to participate in both study stages. The enrolled clinic included 2 physicians, 2 medical office assistants, and a newly hired social worker and registered nurse (RN). The practice used an electronic medical record (EMR) but had no experience with the use of a patient portal, and their EMR did not have an embedded portal. The practice had an identified patient panel within the EMR and received summary patient data from the KB Patient Experience Survey (a questionnaire currently in use by the Divisions of Family Practice to understand patient experience with care) and the Canadian Primary Care Sentinel Surveillance Network [30], a primary care research network that offers a web-based data presentation tool to improve primary health care delivery outcomes across the country. However, these data were not used to practice QI.

One of the initial joint decisions was the identification of the clinical domain of study for this intervention—mental health care. The factors considered in this decision included the limited number of mental health providers in rural primary care, the high prevalence of mental illness [25], and the providers’ current familiarity with and routine use (including at the point of care) of two patient-generated mental health measures—the Patient Health Questionnaire-9 (PHQ-9) depression measure [31] and the Generalized Anxiety Disorder 7 item (GAD-7) [32].

### Co-design Stage 1

#### Overview

This stage involved coproducing the implementation and evaluation plans. The components are shown in Figure 1. Methods for data collection included team mapping, care workflow, portal development, emails, and summary meeting notes. Outputs of this stage included mapping the care team’s roles and responsibilities, identifying relevant PCMs, detailing the office and clinical care workflows to enable PCM use, optimizing the use of the patient portal, and documenting learnings to understand issues and generate solutions.

#### Team-Based Care Mapping

Team roles and responsibilities were mapped using the team mapping method, a facilitated cocreation workshop designed to help groups explore how to work together in a primary care team [33] and informed by the circle of care modeling [34], an analytical technique to develop the PCM adoption methods and create and validate patient use cases. A 2-hour–long team mapping session, using patient personas focused on mental health concerns, was held over Zoom (Zoom Video Communications) with all providers and staff from the clinical practice and members of the research team. The mapping session explored and defined team roles and responsibilities in caring for the simulated patients and how PCMs could support care decisions. This exercise also explored the use of technology, such as patient portals outside of regular visits to engage patients, and workflow associated with monitoring the data. Following the session, facilitators generated a summary report that included images of the patient-centered maps that were created, as well as summaries of the PCM gaps and potential solutions, categorized for discussion with the care team.

#### Patient-Centered Measurements

Discussions with providers led to the following selected PREM and PROMs: (1) a multipart question from the KB Patient Experience Survey that contained subitems about whether someone from the care team talked with patients about difficulties taking care of their health, main health goals and priorities, stressors, needed support, medication review, and offer of preventive care; (2) Patient-Reported Outcomes Measurement Information System Item Bank (version 1.0)—General Self-Efficacy Short Form 4a—a general population measure with demonstrated good convergent validity, internal consistency reliability, model fit, and sufficient unidimensionality [35,36]; (3) PHQ-9 depression measure; (4) GAD-7; and (5) self-action plan for depression [37]. The PHQ-9 and GAD-7 are validated measures already in use by the practice at the point of care.

#### Care Workflow

A group session, held with the clinic after the team mapping exercise, focused on reviewing the mapping report and how PCM gaps could be addressed through alternative workflows [38]. An electronic patient-reported outcomes toolkit [39] guided discussions related to improvements in office efficiency and integration of electronic PGD. Iterations of care workflow were brought forward as a flow diagram for feedback at the next meeting. In consultation with patient partners and co-designed with the care providers and clinic staff, an initial workflow was identified for the implementation stage.

#### Patient Portal

The research team selected a commercial vendor portal that had a patient-centered perspective (eg, patients could select who they want to share data within their care team). The web-based portal was used as a stand-alone system during the study (ie, was not interoperable with the clinic’s EMR system) as no current tethered or interoperable patient portal was available for use in this study, and the industry partner was amenable to adding selected PCMs and tailoring to study needs (eg, education and resources). Ongoing adjustments to the user interface were gathered and provided to the industry partner for implementation.

The portal had a user interface for patients and a separate one for the clinic staff and care providers. The researchers had access to both the interfaces. Of the 7 functionalities assessed in a recent Cochrane review on patient access to EMRs [40], this portal provided for PCM, including tracking, education, and a reminder feature that was sent if a requested patient questionnaire was not completed within 3 months. The portal did not provide the ability to request other information, bidirectional communication and sharing, or the ability of patients to manage their care. The materials provided in the portal included selected PCMs and educational resources.

### Preparing for Implementation and Evaluation

Preparing for stage 2 encompassed patient recruitment, change management support, portal deployment, and development of the evaluation.

### Recruitment of Patient Participants

Clinic staff and care providers contacted patients who had been living with a mental health diagnosis for many years to inform them about the study. Interested patients were provided with a flyer that included contact information for a researcher (MA) who provided study information and a consent form before enrollment. Four patients agreed to participate in the study. Participation involved using the patient portal over 4 months and interviews to discuss how PCM and educational materials within the portal were used during their care. Owing to the COVID-19 pandemic, most clinical visits between patients and providers were held over phone.

### Implementation—Stage 2

This stage comprised activities to operationalize the intervention and adapt design components based on iterative feedback. The components of this stage are shown in Figure 1.

####  Patient Workflow and Portal Use

The use of the portal was presented to patients at the start of the intervention as a part of their workflow. Iterative patient workflow adaptations were made based on patient feedback during the implementation and evaluation stages.

One researcher (MA) provided training in portal use for all patients, clinic staff, and care providers. The patient user interface allowed patients to see messages from their provider, complete the PREM and PROMs, and explore educational materials. The portal was set up to send an email to inform patients that there was an invite in their portal account. Both patient and provider portal interfaces allowed completed PCMs to be viewed on the computer device screen or downloaded as a document that clinic staff could upload into the patient’s record in their EMR. Patients and providers could also view data trends of these measures (eg, scores on PHQ-9) if more than one was completed.

#### Care Workflow for Providers

The care workflow, finalized in the previous stage, was used as the initial care workflow for the study implementation. Individual discussions with providers and clinic staff during the intervention focused on changes to the care workflow specific to care pathways such as screening, monitoring, and follow-up resulting from the addition of PCM to team-based care processes. For example, the RN took responsibility for sending PROMs and educational materials to patients biweekly to complete through the portal. The PREM was sent at the beginning and end of the portal-use period.

We recognized that because of the lack of interoperability between the portal and EMR, we would have to simulate some embedded portal functions, such as sending reminders and data entry. For example, the portal-completed PROMs were intended to be reviewed during clinic visits. To achieve this, the RN and a researcher (MA) shared responsibility for checking for upcoming appointments, deploying the PROM and checking for completion and transferring the PROM score into the EMR encounter notes section, attaching the document, and entering the score into the existing PROM sections of the EMR. The researchers met periodically with the RN to adjust the implementation efforts.

### Evaluation Plan—Stage 2

#### Interviews

A general interview guide approach was used for the patients (Multimedia Appendix 1) and providers (Multimedia Appendix 2). Patient interview questions included interactions with the portal, use of questionnaire results and educational materials by themselves and with the provider, and how other tools could be used to complement communication with the portal. Interviews were scheduled within 1-5 days after a virtual or phone visit with their provider. A total of 13 patient interviews with 4 patients were conducted, and the number of interviews with each patient was dependent on the number of care visits (2-4 visits) during the study period. Provider questions included visit information, use of patient questionnaires during the visit, whether educational materials prompted discussion, and whether the questionnaire results/educational materials influenced the visit or team interactions. An individual interview was conducted with all 4 care providers and 2 clinic staff members at a convenient time following a patient visit (timing varied between 1 week and 1 month). To gain immediate insights, a web-based survey was provided to physicians immediately after a patient visit (Multimedia Appendix 3).

All interviews were conducted virtually (phone or video) by two researchers (SD and MA) and lasted 10 to 30 minutes. Both researchers had experience with qualitative research. Notes were taken by both interviewers and combined as summary notes. The summary interview notes were coded by one researcher (MA) using ATLAS.ti (ATLAS.ti Scientific Software Development GmbH), and the coding reports and original data were reviewed by 2 additional team members with experience in the analysis of qualitative data (MS and SD). The final interview coding reports were confirmed by the research team.

#### PCM Data Analysis

The framework method for the analysis of qualitative PCM data was used. The framework method applies a matrix structure to facilitate the recognition of patterns and has been used effectively under the leadership of experienced qualitative researchers [41]. Data analyses involved looking across interview coding reports, team mapping reports, portal use, and care workflow diagrams to determine areas of convergence or divergence and develop a narrative of the evaluation of the implementation. Our reflective practice comprised the development of new care workflow diagrams, iterative writing, and discussion among the research team at monthly analysis meetings.

## Results

### Co-design—Stage 1

The collective results of stage 1 were used to inform stage 2.

#### Team Mapping to Inform Care Workflow

**Figure 2:**
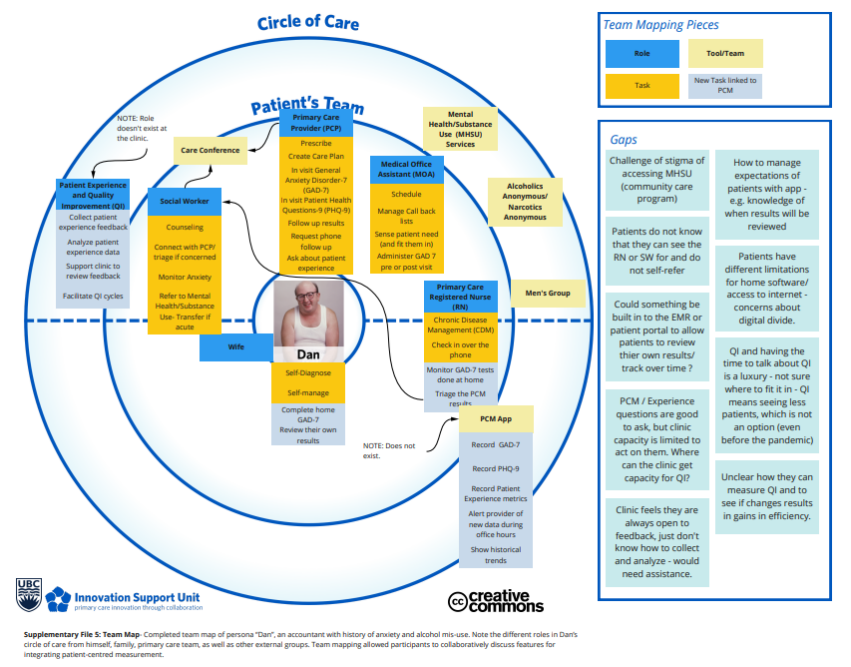
Mapping team-based care for patients with mental health issues.

The team mapping exercise focused on two areas: care of patients with mental health concerns and how PCM could be used in practice. The session produced *circle of care* maps with defined team roles and responsibilities in caring for the simulated patients (Figure 2). The session’s discussion focused on PCM integration and how PCM could influence care.

**Figure 3:**
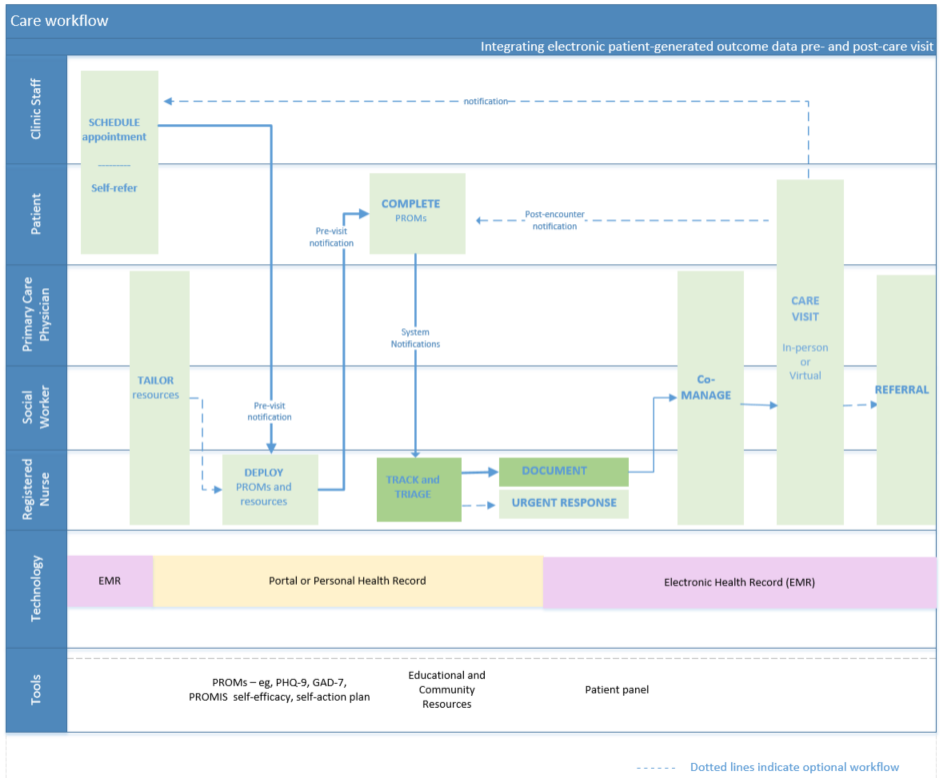
Final team-based care workflow with electronic patient-generated data [42]. GAD-7: Generalized Anxiety Disorder 7-item; PHQ-9: Patient Health Questionnaire-9; PROM: patient-reported outcome measure; PROMIS: Patient-Reported Outcomes Measurement Information System.

Following the mapping session, co-designing efforts produced an initial care workflow for incorporating PREM and PROMs into routine care, before, during, and in between a patient’s clinic visits, using a patient portal. Five key activities were identified: (1) deploying PCM, (2) collecting electronic patient data, (3) tracking completion, (4) reviewing data, and (5) documentation. Workflow changes were then designed to incorporate these activities, and a final workflow diagram was developed (Figure 3 [42]). Before the visit, the patient or clinic staff may initiate a visit appointment, the care team may tailor resources and PCM questionnaires in the portal, the patient would receive notifications and review the resources, and then complete the questionnaires. The nurse would track and triage the PCM scores and initiate urgent responses if needed. During the virtual or in-person visit, the physician might review the PCM scores with the patient, document appropriate actions, and refer the patient to other providers as needed. In between visits, the care team would comanage the patient.

#### Strategies and Concerns About Integrating PCM

On the basis of team mapping and care workflow sessions, we constructed Table 1 with learnings and potential solutions to care gaps and team limitations, strategies needed to allow the use of PCM, constraints in the current workflow including restrictions because of the COVID-19 pandemic, and modifications needed to the patient portal to enhance care. Providers commented that the mapping and workflow sessions mirrored clinical practice well. The major concerns expressed included the practice’s limited capacity for incorporating PCM gathered outside the clinic visit (both electronic and paper-based), patient expectations for review of their data, and the provider’s knowledge and clinic capacity that limited engagement in QI. Providers expressed concerns about the rural context with lack of access to high-speed internet, insufficient resources to manage practice workload, and current fee-for-service structures that did not provide an allowance for the collection and use of PCM.

**Table 1:** Stage 1—gaps, learnings, and potential solutions before implementation.

Co-design step	Learnings	Potential solutions
Team roles and responsibilities	Patients do not know about roles or access to a RN^a^ or social worker No individual was trained in using data for QI^b^ The stigma around mental health care and access to community program No ability to track medications or change in PGD^c^ scores	Information about staff and other resources in portal Engage with training resources (eg, future staff training) Improve connections so these are part of team Expand team: pharmacist
Strategies (what needs to be in place to use PGD)	Patients and providers need access to data and tracking; best if integrated Patients may need reminders: bring data or trigger discussion Increase the level of patient engagement	Make PGD available through the portal; EMR^d^ linkage; explore ways to document Consider reminders in the portal Provide resources in the portal
Care workflow	No capacity or training to use patient experience data for QI COVID-19 pandemic restrictions prevent hallway conversations There is no way to trigger follow-up (eg, significant change in PGD score)	Begin with joint selection of an experience measure Schedule brief virtual work huddles at the start of each day Plan to explore options in stage 2
Patient portal	There was concern regarding limited digital literacy There was concern regarding patient expectations for review of data Patient knowledge gaps around depression and anxiety	Patient interview data to understand needs Careful use of language and messages to patients on expected follow-up Add educational resources to portal and delivery plan

^a^RN: registered nurse.

^b^QI: quality index.

^c^PGD: patient-generated data.

^d^EMR: electronic medical record.

#### Customization of the Patient Portal

**Figure 4:**
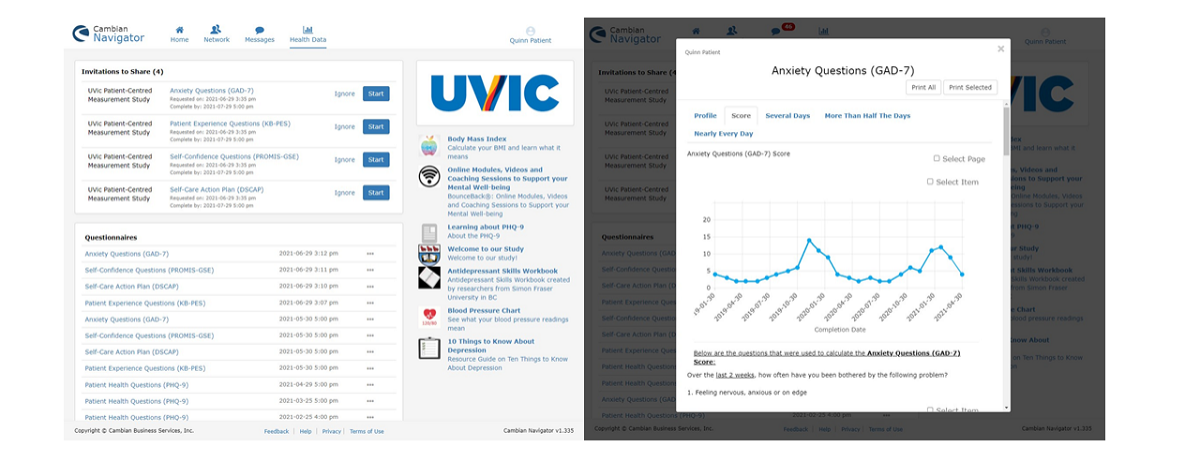
The patient portal—patient-centered measurement questionnaires, resources, and functions.

The patient portal was collaboratively prepared with selected mental health questionnaires, patient experience measures, depression self-action planning tool, and educational resources, and comprised a feature for trending data such as the anxiety questionnaire, GAD-7, in Figure 4. An appraisal guide was created to select the educational and resource material to ensure that the material was evidence-based, used patient-oriented language, and was adaptable to local contexts (eg, local community resources). Examples of the educational materials are provided in Multimedia Appendix 4.

Before deployment, the portal underwent initial testing by patient partners and an undergraduate student who simulated the role of the patient. Through discussions with the practice and research team, the type, timing of deployment, quantity of educational material, messaging, and notifications within the portal were determined before launching portal use.

### Implementation and Evaluation—Stage 2

#### Care Pathways and Portal Use

**Figure 5:**
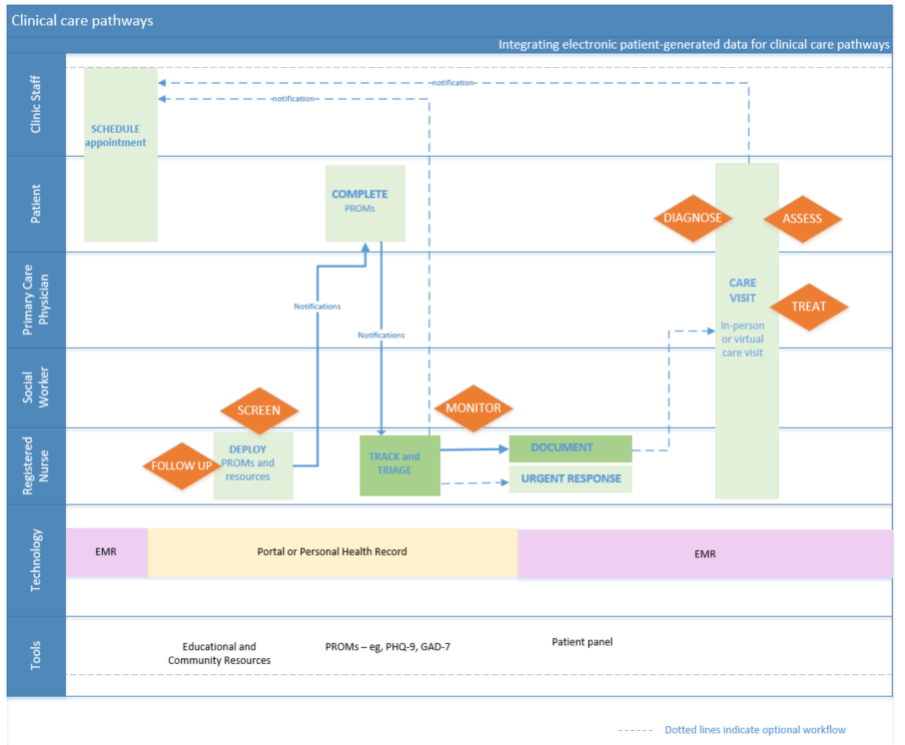
Clinical care pathways with electronic patient-generated data [42]. EMR: electronic medical record; GAD-7: Generalized Anxiety Disorder 7-item; PHQ-9: Patient Health Questionnaire-9; PROM: patient-reported outcome measure.

On the basis of workflow discussions with care providers and patient partners, a clinical care pathways diagram was produced (Figure 5 [42]) to identify the opportunities for care team members to integrate PCM (eg, asynchronous screening or follow-up or during an encounter). There are 7 PCM-related clinical care activities performed by different care team members to screen, triage, assess, diagnose, treat, monitor, and follow up with the patient, which can be guided by their PCM data. Nurses and social workers may initiate screening, monitoring, and triage of patients, or when triggered by web-based notifications and reminders from the portal. Physicians may refer to PCM data when assessing, diagnosing, and treating the patient during an in-person or virtual visit. In between visits, the nurse could follow up with the patient to provide resources and PCM questionnaires within the portal, monitor for completed PCM data, and schedule notifications as reminders to complete them.

**Figure 6:**
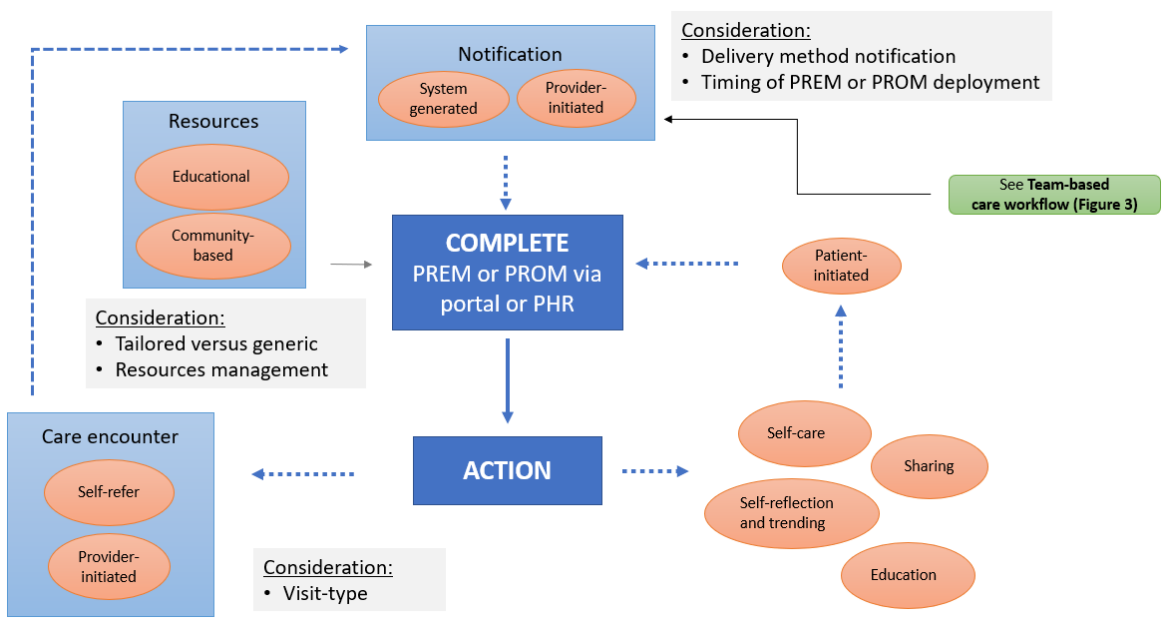
Patient workflow with electronic patient-generated data [42]. PHR: personal health record; PREM: patient-reported experience measure; PROM: patient-reported outcome measure.

A patient workflow based on patient feedback was created to incorporate real and potential actions related to PCM (Figure 6 [42]). PCM-related activities for patients are to review resources in the portal and evaluate their relevance, respond to notifications, complete PCM questionnaires indicated by the provider or on their own initiative, and take actions in response to new learnings. These actions include reflecting on their condition in response to new information, initiating self-care, tracking trends over time, sharing information with family or care network, reviewing additional resources, following up with the care team, and preparing for a visit. Considerations included delivery-method notification, the timing for completion of questionnaires and resources, tailoring efforts of educational materials, and visit type (in-person or virtual). A video was created [43] to illustrate study results relating to the use and integration of PGD into clinical care from the patient’s perspective.

Table 2 provides excerpts from the summary notes that demonstrate our key learnings from stage 2. The qualitative synthesis of 13 patient interviews indicated that the portal was easy to use and valued. Two patients noted the inability to use the portal during times of severe depression and fatigue. Providers saw benefits in patients’ use of the portal for information and to give them a sense that care was nearby. The major problems identified with provider use of the portal were lack of portal integration with the EMR and lack of alerts for changes in questionnaire scores that should trigger action. For providers, to limit the extra work across the team, 1 team member (RN) oversaw the PCM data in the portal and transferred it into the EMR. Even with the simulated integration of data transfer into the EMR by the RN, the process of reviewing those data elements in the EMR was often overlooked during a care visit.

All patients read at least some of the educational material provided, with skill-building workbooks being the most appreciated. One patient stated that “knowledge of our illness is vital to taking steps to live with illness and attain victory as often as possible.” Getting information over time rather than all at once was useful to avoid overwhelming the participant. Most patient participants had been living with a mental health diagnosis for many years and reported that the initial educational materials offered would have been helpful earlier in their diagnosis and illness. At their request for more advanced information, patient-friendly abstracts of scientific papers were added to the portal. Patients also wanted the ability to rate articles for their usefulness. Providers appreciated the potential for educational resources to be tailored to the patient’s stage and needs. However, they recognized that they would not have the resources or domain expertise needed to sustain ongoing educational material development or deployment in the portal.

**Table 2:** Stage 2: learnings from patient-centered measurement (PCM) and portal implementation.

Learnings	Patients	Providers
Use of patient portal (not used during visits)	View educational and community resources and complete PROMs^a^ and PREMs^b^ A way to overcome isolation and focus on self Unable to use during times of severe depression or fatigue	Ways to provide information to the patient and impart “a feeling that care is nearby” A lack of integration with EMR^c^ Resulted in use by a single nurse to manually transfer patient-generated data, which were rarely reviewed during a care visit Lack of alerts to trigger action reduced portal usefulness
Value of educational resources	Knowledge is vital to overcoming illness Provide credible information and avoid getting “lost in the abyss” The initial educational materials provided would have been helpful earlier in their diagnosis and illness. Personalized material would be valuable	Appreciated the potential for educational resources to be tailored to the patient’s stage and needs “Not able to sustain ongoing educational material development or deployment”
Use of PCMs	Completing PROMs was extremely “validating” Sense of being heard and capturing more of the relevant information about their mental health Tracking and trending scores using a portal “painted a picture of where I am” Frustration that their providers were not asking about or reviewing PROMs during care visits Unclear who was to bring up the PROM	Did not use within portal because of the following reasons: Lack of integration of portal with EMR (only total score of PROMs were manually entered into the EMR) Lack of alerts for changes in questionnaire scores that should trigger action Belief that the PCM did not add to the existing relationship
Optimizing completion and use of PROMs	Timely reminders for PROM completion PROMs needed that address function to aid “what created my responses” as part of the interpretation of a measure	Timely customizable reminders for PROM completion Interoperability of systems Additional training on use of PROMs

^a^PROM: patient-reported outcome measure.

^b^PREM: patient-reported experience measure.

^c^EMR: electronic medical record.

#### PCM Use in Team-Based Mental Health Care

Three patients reported that the process of completing PROMs was extremely *validating* in that they learned more about themselves and gained confidence in reaching out for support. As one patient noted on reviewing the PHQ-9 with a resident physician, the experience was “unexpectedly rewarding and validating (same page together) and resulted in a treatment change.” Several patients noted that clinic visits in their current form are often used to revisit past appointments or begin with general questions that may not relate to a patient’s current status. As 1 patient, who was hesitant to talk with the physician about anxiety, noted, completing the questionnaire ensured that all pertinent aspects were covered, preventing communication disconnect. In addition, tracking scores were appreciated; 1 patient stated that the weekly trend “painted a picture of where I am.” However, 1 patient noted difficulty in interpreting depression scores in that they did not provide insight into “what created my responses.” Suggested additions by patients for PROMs included functional measures (eg, what is working now for or against you), more sophisticated graphics for tracking scores and more specific questions in the action plan based on depression scores. Patients wanted to be able to complete PROMs on their own and trend data over multiple years.

Patients expressed frustration that their providers did not review PROMs and did not ask about PCM at visits. One patient reported that she thought she was doing something wrong in not having questionnaire data at her visit. Providers noted that although PCM collected before a visit could make visits more efficient, they did not review them for reasons noted in Table 2.

As PGD in the portal was a new activity, there was also a lack of clarity when the PROM scores would be viewed and discussed. Patients were used for in-person visits and thus did not consider opening the portal during their virtual visit. Each provider had different preferences for where to view these data but had not established a practice of viewing them. The limited-time for visits meant that providers prioritized the purpose of the visit and planned to look at the PROM scores during the patient’s annual mental health check-up.

Concerning optimizing the use of PCMs, both patients and providers wanted to be able to set timely reminders for PROM completion. Patients sometimes logged into the portal to see an invite and start completing a PROM, and then realized that they wanted to return when they could give more focus or were less fatigued. The lack of reminders meant that patients often forgot to return and complete PROM. Similarly, providers wanted the ability to customize reminders (eg, 24 hours before a visit or a week after the initial invite). Providers also noted that they lacked education and training on PROMs’ limited use, including interpretation of a PCM score and what constituted a significant change.

#### Quality Improvement

**Figure 7:**
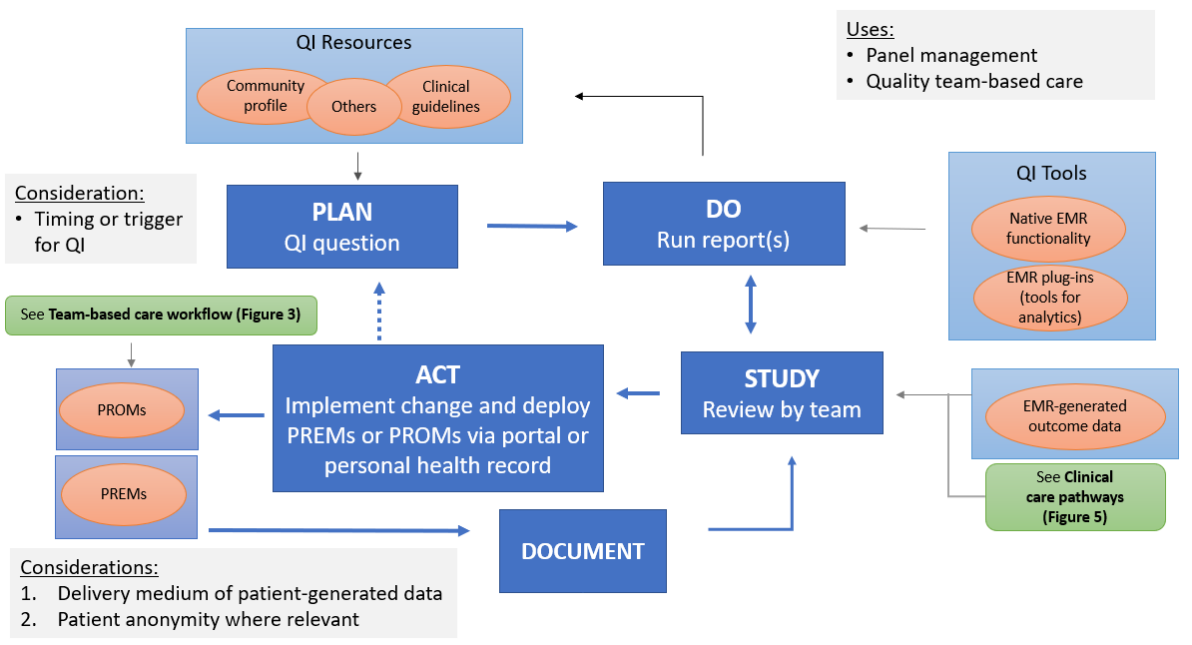
Quality improvement (QI) in team-based care workflow with electronic patient-generated data [42]. EMR: electronic medical record; PREM: patient-reported experience measure; PROM: patient-reported outcome measure.

Although QI activities were never completed, discussions were held to explore opportunities within workflow and care processes to carry it out. Examples of mental health care included PHQ-9 completion, counseling, and medication prescriptions for patients with a diagnosis of depression. The resultant QI process workflow is displayed in Figure 7 [42]. PCM-related QI activities for the care team are based on the plan-do-study-act cycle and documentation processes that can be informed by PCM. Planning is driven by clinic priorities and capacities and is further guided by community health profiles and the latest clinical practice guidelines. Doing involves running patient panel queries using the clinic EMR and related external databases or applications to identify areas for attention. Studying, acting, and documentation involves a detailed review of the panel query outputs, implementing specific actions, and documenting the actions and results when available. The PCM focus brings awareness to specific groups of patients that may require action depending on their health situation and PCM scores.

To date, most QI efforts have been focused at the regional level, and the clinic does not know how to apply PREM results to clinical processes with multiple providers. An agreement was received around its value, but resistance was observed, and one provider commented on the lack of medical school training in QI. There was also a sense of technology overload, where providers had to remember multiple login codes and how to view data across more than 4 different systems. QI tools and resources available, delivery medium and type of PCM, timing and triggers for QI, and maintenance of patient anonymity when using PREMs were discussed.

#### Additional Learnings

Knowledge sharing (or IKT) throughout the study was invaluable to our process and learnings. Specifically, during all interactions and interviews with knowledge users, the researchers received productive feedback to questions like *how we are doing* and whether *we hit the mark* with iterations of (1) process descriptions; (2) design planning, implementation, and evaluation actions; and (3) documentation of identified issues and solutions.

Although providers noted that it takes a *whole team* to care for patients with mental health disorders, clinical resources were overwhelmed and there were limited community mental health services in this rural area. Multiple patients commented on how their physician was overworked, so they were mindful of what could be done at a visit, although they would have preferred mental health visits more frequently than once annually. The addition to the practice of the social worker was relatively recent, and one patient did not know about the availability of the RN or social worker or how they could fit into their care. Another patient stated that they wanted a physiotherapist to be part of their team. Both patients and providers noted that rural living was associated with fewer specialist resources and longer wait times.

Since the COVID-19 pandemic, virtual care visits have become a routine part of practice. One staff member noted that patients were no longer in the waiting room or available to complete questionnaires on-site. Both patients and providers felt that virtual care was less valuable because of lack of personal contact; one provider stated that “the loss of human connection is devastating.” One patient reported that they might have brought up their anxiety if face-to-face and that being virtual missed the seriousness of their illness. However, patients also preferred to answer PROMs at home, as the answers seemed more honest than completion in a public waiting room. One clinic staff member noted a possible benefit of phone visits in a rural (small population) clinic in that patients could have a degree of anonymity by not having a face to recognize when seen out in the community.

## Discussion

### Principal Findings

IKT offered the research a continuous and rich understanding of patient and provider needs and current challenges in the context of team-based care and PCM, as well as potential best practices for integrating PGD using a patient portal. Providers were unable to incorporate the use of electronically generated mental health PROMs within team-based care during this study for several reasons including workload, need to prioritize issues to address during an encounter, lack of easy access to the data, lack of value placed on the data, and lack of education about and practice in its use. The absence of established provincial standards for interoperability between systems hampered the integration of electronic PCMs in team-based care. In a review of personal health record functionalities and implementation, Harahap et al [44] identified interoperability as a key implementation issue, as well as security and privacy, usability, data quality, and personalization as other important factors. Digital health transformation at the system level is needed to realize interoperability, providing standard definitions for data exchange and cooperation with the patient, provider, and organizational systems. Using an ontological information model, Plastiras and O’Sullivan [45] demonstrated the feasibility of transferring PGD using common standards from personal health records to EMRs. Interoperability must be addressed to ensure that electronic PROMs and PREMs from patient systems can be fully integrated within provider systems and readily available at the point of care. In addition to the issue of interoperability, the authors perceived that the limited experience of these newly formed primary care teams, and having to shift to virtual care during the pandemic made it less likely that any member could ensure delegation and use of the data provided.

Solutions to learnings from the co-design step that were acted upon (ie, engaging with the local Division of Family Practice around QI, patient reminders for the completion of questionnaires, research assistant tracking, and providing patient education and resources within the portal) were valued and demonstrative of the way forward. Ultimately, the gap in getting data viewed in the EMR and usable at the time of a visit was not accomplished. For digital tools to be successful in addressing provider workload, authors of a systematic review [40] identified a need for training, reducing documentation and task time, expanding the care team, and leveraging QI processes in workflows.

Although QI was an intended part of PCM advancement, it was discovered early in the study that providers felt that the practice could not engage in practice improvement using PREMs. An additional knowledge user with subject-matter expertise in QI was added to the team to present information on EMR panel report analysis, community profile, best practices, and clinical practice QI guidelines using PROMs. Although providers expressed interest in the future use of PCM for QI, because of the COVID-19 pandemic, frustrations expressed over prior poor quality QI data provided, and inexperience with use, the study shifted to provide an opportunity to explore QI and educate the practice on ways PCM could be used for QI. The QI activities were not implemented or evaluated.

PREMs are being collected through population surveys in the geographic area of this study, but QI efforts are still nascent, and there is no single best way to collect or use PCM for QI. Gleeson et al [46] systematically reviewed current QI efforts using PREMs and found that most practices attempted small, incremental changes to services that did not require a change in provider behavior and resulted in unclear impacts. They called for more attention to how PREM data can be used to inform practice changes that have a positive influence on the patient care experience. Translating new ideas into practice among early-career providers requires three considerations—credibility, practicality, and need [47]. Efforts to incorporate QI into routine practice will likely require attention to messaging and creating digital solutions to address these issues. In addition, simply providing PGD, even if entered into the EMR, has the potential to increase provider burnout. A review of health record-integrated PCM found that technostress (technological complexity, uncertainty, overload, insecurity, and invasion [higher patient expectations]), time pressure, and workflow-related issues need to be addressed to accelerate the integration of PCM into clinical care [48]. Therefore, future endeavors will need to consider the human and fiscal resources needed for QI in clinical practice and the integration of PGD into the digital health ecosystem.

To create primary care team-based practices that value and use PCM to improve care, a culture change is needed. This process, in the experience of one of the authors (MS) in teaching evidence-based medicine and research training, takes 3 to 5 years, with time for training and practice through targeted, small-scale projects. For example, the introduction of QI training of multidisciplinary teams in local health departments in North Carolina resulted in small but important changes in organizations’ cultures over 5 years, increasing engagement in future QI, and improving overall care and services [49]. Mandating QI may be problematic, with a before-and-after study of Foundation Year 1 doctors in the United Kingdom reporting less motivation to complete QI projects and placing less importance on QI for their professional development despite a significant increase in overall QI knowledge at the end of the year [50]. It remains to be seen if the BC support system approach to practice facilitation for QI is successful in this effort.

However, patients found the process of completing and tracking the results of mental health-related questionnaires on portal validation useful in capturing relevant aspects of their experiences, a unique feature of this study. The importance of portals as a communication tool and limited use of PGD was noted in an umbrella review [51], and lack of bidirectional communication was likely an additional barrier to PCM used in this study. Patients commented on how PROMs could provide a way to focus visits on what is of the greatest value for them at the moment. PCM may be particularly relevant for patients with illnesses for which there are no biomedical markers. For people living with mental health concerns, these measures can aid in communication when the illness makes it difficult to communicate the severity of their difficulties. Although evidence supports the use of patient medical home care models that include data-driven quality of care and patient engagement [2], what seems to be missing is the value placed on PCM by providers and an understanding and appreciation of ways in which the use of these data can advance communication, care quality and QI. To achieve the patient-centered potential of PCM requires a conceptual shift in workflows, where patients and providers are encouraged to bring PGD, particularly around function, into care discussions.

### Study Limitations

As this was an exploratory study within a single clinic, the transferability of the findings to another context needs to be explored further. The study was carried out in a rural, early adopter patient medical home practice with a small care team and few patient participants, which may not be representative of other primary care models, larger clinics, or urban settings. As the study focused on mental health, the relevance of our findings to other health conditions is limited.

The researchers supported transferability by providing a detailed description of the context and location, and trustworthiness by being transparent about our methods for data collection and analysis. The study was conducted during the pandemic, which made recruitment of care teams and patients particularly challenging. We strove to address the limited sample size by adding richness to our data through multiple study methods and an iterative process of knowledge sharing between researchers, including patient partners and knowledge users. Being a transdisciplinary team of patient partners, providers, and researchers across multiple disciplines enabled us to bring in multiple perspectives during the analysis.

Limitations were introduced during the data collection and analysis. The persona image was selected to reflect how Dan may be dressed in an examination room. Upon reflection, we realize that Dan’s appearance would be quite different during a virtual visit and would have selected a persona image that does not reinforce power differentials or stereotyping [52]. Although we did not record interviews, we used 2 interviewers at each session, and summative interview reports were iteratively compared with all PCM study data by the research team. Memos in the summary notes were used to record when the interviewers had different interpretations. Each researcher established their process for reflection, and we did not have a standard way to record and review individual reflections.

### Conclusions

The value of PGD and the need for PCM methods to collect, integrate, and use PGD in team-based care remain challenging. Through collaborative and adaptive efforts, this gap was narrowed by demonstrating ways in which PGD can be incorporated into clinical practice within a Canadian team-based primary care setting. The conceptualization of PCM methods to accomplish this goal is well served by an IKT approach. IKT offers a beneficial technique for addressing our knowledge of users’ needs related to the collection, integration, and use of PGD, bridging the knowledge to action gap and setting the stage for future success.

## Appendix

Multimedia Appendix 1 Interview guide and questions for patients.

Multimedia Appendix 2 Interview guide and questions for providers.

Multimedia Appendix 3 Questions for providers—web-based survey.

Multimedia Appendix 4 Examples of educational resources made available on patient portal.

## Abbreviations

BC: British Columbia
EMR: electronic medical record
GAD-7: Generalized Anxiety Disorder 7-item
IKT: integrated knowledge translation
KB: Kootenay Boundary
PCM: patient-centered measurement
PGD: patient-generated data
PHQ-9: Patient Health Questionnaire-9
PREM: patient-reported experience measure
PROM: patient-reported outcome measure
QI: quality improvement
RN: registered nurse
