# Supplementary material for: Paving the Way for Electronic Patient-Centered Measurement in Team-Based Primary Care: Integrated Knowledge Translation Approach
Source: JMIR Form Res. 2022 Mar 18;6(3):e33584. doi: 10.2196/33584 (PMC8976252; doi:10.2196/33584)
Supplement: Multimedia Appendix 1 [file formative_v6i3e33584_app1.docx]

**Multimedia Appendix 1: Interview guide and questions for patients**

General:

Review of informed consent and what is involved for today

- - Do not have to answer any questions you don't feel comfortable answering.
  - Everything will be kept confidential, and we will share the results with the clinic, but your name will not be referenced.

**First interview** (adapt as needed for second++ interview)

Questions:

1. How have you been finding the portal so far?
2. What type of visit was this? (i.e. virtual, phone, face-to-face). Who was the visit with (e.g. doctor, nurse, social worker)? When was your visit? When did you complete the questionnaire (e.g. before the visit, during the visit)?
3. Can you walk me through how the questionnaires you completed were talked about in the during the visit? Did you notice any change from previous visits?
   1. How was it referred to (e.g. looked at specific questions, reviewed document together, or in a general discussion)
   2. Who referred to it (i.e. doctor, nurse, social worker, medical office assistant)?
4. Did you and your care team refer to the information that you entered through the portal?
   1. Prompt: did it change the discussions you had with your provider?
   2. Prompt: did it help in deciding on treatments or next steps?
5. How did you like the additional articles that were provided through the portal?
   1. Prompt – were they useful, informative, not very helpful
   2. If helpful – which materials did you find most useful?
   3. If not helpful – what would have made these more helpful?
6. Did the questionnaires you answered through the portal support your care decisions?
   1. If yes, in what way?
7. What did you find worked well in the portal? What didn't work well?
8. How would you rate your comfort level with technology?
   a. how often do you use the internet? quite a bit? a little bit?

Closing Information:

Next steps: We are going to start adding in articles that you should receive each week. We will also be adding two more questionnaires that the clinic may send to you. We are interested in how your finding them, whether they are helpful or prefer other types of articles. If would like more or less articles, etc. There is also a questionnaire that will be sent out that asks about your experience with the clinic. This one is not shared directly with the clinic.

**Second++ interview**

Adapt questions 1-8, as relevant, PLUS

Questions:

1. Did you use the questionnaires to support your health over time?
   1. Prompt: Did you use them to track your symptoms or response to treatment?
   2. Do you think completing the questions online were of benefit?
      1. If yes, in what way – Prompt: more honest, more efficient use of time
2. What did you find worked well in the portal? What didn't work well?
3. If this technology (or a similar one) was available through your doctor’s office, would you continue to use it? Are there other features or functions of the portal that you might like to have (e.g., weight or blood pressure tracking, other educational materials, links to support groups)?

12. How would you rate your comfort level with technology?
