# Supplementary material for: Paving the Way for Electronic Patient-Centered Measurement in Team-Based Primary Care: Integrated Knowledge Translation Approach
Source: JMIR Form Res. 2022 Mar 18;6(3):e33584. doi: 10.2196/33584 (PMC8976252; doi:10.2196/33584)
Supplement: Multimedia Appendix 2 [file formative_v6i3e33584_app2.docx]

**Multimedia Appendix 2: Interview guide and questions for providers**

General:

Review of informed consent and what is involved for today

- - Do not have to answer any questions you don't feel comfortable answering.
  - Everything will be kept confidential, and we will share the results with the clinic, but your name will not be referenced.

Questions:

1. How did you use the technology? (Prompt: collected information from the questionnaires, sent educational material to the patients?)
2. Can you walk me through how the questionnaires were documented in the patient's record? Who on the team reviewed these questionnaires? How were the questionnaires used during a patient visit?
3. Were the questionnaires used to support quality improvement or management of patient populations?
   1. If yes, how did you use the information?
4. Did you use the technology to track trends?
   1. If yes, how did this work (Prompt: addressed at the time of a visit, used to provide outreach to the patient based on risk score)
5. How did the workflow change in the clinic with the introduction of the technology-supported questionnaires?
6. What were some barriers in using the questionnaires that patients provided through the technology?
   1. Prompt: created extra work (and for whom), workflow issues around having someone gather and record the information, trust issues about data accuracy.
7. Did the technology facilitate discussions with your patients? (Prompt: did you think that you got information that would not have been gathered during a visit?)
   1. If yes, can you tell us about a time when this happened?

**Questions for additional interviews**

How have you been viewing the questionnaires in the portal?

How are the scores used? How you been interpreting the scores for the upcoming visit?

How have you used the scores to triage or have discussions with other people on the clinic?

Have you looked at the reports generated in the trends?

How have you been selecting the articles to be sent out? Is individual or group level?

What kind of reminders would be helpful, if helpful?

How has the workflow changed with the portal?
