# Supplementary material for: Paving the Way for Electronic Patient-Centered Measurement in Team-Based Primary Care: Integrated Knowledge Translation Approach
Source: JMIR Form Res. 2022 Mar 18;6(3):e33584. doi: 10.2196/33584 (PMC8976252; doi:10.2196/33584)
Supplement: Multimedia Appendix 3 [file formative_v6i3e33584_app3.docx]

**Multimedia Appendix 3: Questions for providers** — **web-based survey**

Questions:

| **1)** **Date of visit:** |  | | | | | | |  |  |
| --- | --- | --- | --- | --- | --- | --- | --- | --- | --- |
|  | |  | | | | | |  |  |
| 1. **What type of visit was this?** | |  | Video |  | Phone |  | In-person |  |  |
|  | |  |  |  |  |  |  |  |  |
| 1. **Who was involved in the visit?** | |  | Doctor |  | MOA |  | Nurse |  | Social worker |

1. **When was the questionnaire(s) completed?**

|  | Not completed for the visit |  |
| --- | --- | --- |
|  |  |  |
|  | Prior to the visit by the patient without any prompting by the clinic |  |
|  |  |  |
|  | Prior to the visit, following a reminder by the clinic |  |
|  |  |  |
|  | During the visit |  |
|  | If during the visit, what steps were taken to get the questionnaire completed? |  |
|  |  | |
|  |  | |

1. **Which questionnaires were reviewed during the visit?**

|  | GAD-7 |  | PHQ-9 |  | PROMIS Self-efficacy |  | Depression Self-action plan |
| --- | --- | --- | --- | --- | --- | --- | --- |

1. **What portal information was referenced during the visit?**

|  | Overall score of questionnaire |  | Specific questions in the questionnaire |  | Changes in score/trend |  | Educational material |
| --- | --- | --- | --- | --- | --- | --- | --- |
|  |  |  |  |  |  |  |  |

1. **Did the questionnaire(s)/educational articles prompt or improve discussion, help with charting, or assist in care decisions with the patient?** (Other examples may include change in medication, management, timing of next visit, link to community supports, education and guidance).

|  | No |  | Yes | If yes, how so? |  | |
| --- | --- | --- | --- | --- | --- | --- |
|  |  |  |  | |  |  |
|  |  |  |  | |  |  |

1. **How did the questionnaire(s)/educational articles impact the following:**

| **a) Efficiency of clinic work** |  | Increased |  | No change |  | Decreased |
| --- | --- | --- | --- | --- | --- | --- |
|  |  |  |  | | |  |
| **b) Team interactions** |  | Increased |  | No change |  | Decreased |
|  |  |  |  |  |  |  |
| **c) Duration of visit** |  | Increased |  | No change |  | Decreased |
|  | | | | | | |

| **9) What other effects were introduced through patient’s use of the portal?** |  |
| --- | --- |
|  | |

| **10) Overall, how was the portal helpful or unhelpful?** |  |
| --- | --- |
|  |  |
