# Supplementary material for: Paving the Way for Electronic Patient-Centered Measurement in Team-Based Primary Care: Integrated Knowledge Translation Approach
Source: JMIR Form Res. 2022 Mar 18;6(3):e33584. doi: 10.2196/33584 (PMC8976252; doi:10.2196/33584)
Supplement: Multimedia Appendix 4 [file formative_v6i3e33584_app4.docx]

**Multimedia Appendix 4: Examples of educational resources made available on patient portal**

| **Informational**   - About the Portal (e.g., Introduction to Cambian Navigator, Getting started) - About Patient-Generated Data and Specific Questionnaires (e.g., Introduction to patient-generated data, How questionnaires are shared) - About Team-based Care (e.g., Role of Social Worker and Nurse [links]) - About Internet Resources (e.g., link to Simon Fraser University site that introduces people to common online technologies) - About Community and Mental Health Resources (e.g., List of mental health resources compiled by Interior Health) |
| --- |
| **Educational**   - General Health Articles (SMART goals, MedlinePlus, Cochrane Library [lay abstracts]) - Articles on Depression and Anxiety* (e.g., What is depression, 10 Things to Know About Depression, COVID-19 impacts) - Internet Links to Resources (e.g., Canadian Mental Health Association, Healthlink BC [includes action plans], BounceBack [online modules], HEADSUPGUYS) - Workbooks (e.g., Antidepressant Skills Workbook, Cognitive Behavioral Interpersonal Skills Manual) - Screening tools (e.g., PhQ-9, GAD-7, KB Patient Experience Survey) |
| **Community Resources**   - Introduction to Boundary Family Services, KB FETCH, Interior Health - Divisions of Family Practice (e.g., Rapid Access to Consultative Expertise, Pathways) |

*Some written by the research team and other’s from publications
